# Supplementary material for: A network dynamical simulation model for the study of antibiotic resistance in microbial communities
Source: Gut Microbes. 2026 Sep 19;18(1):2734703. doi: 10.1080/19490976.2026.2734703 (PMC13596919; doi:10.1080/19490976.2026.2734703)
Supplement: Supplementary file 1 [file KGMI_A_2734703_SM9053.zip › supplementary_file_1/Figures/er_pct_delta_n_edges_n100.pdf]

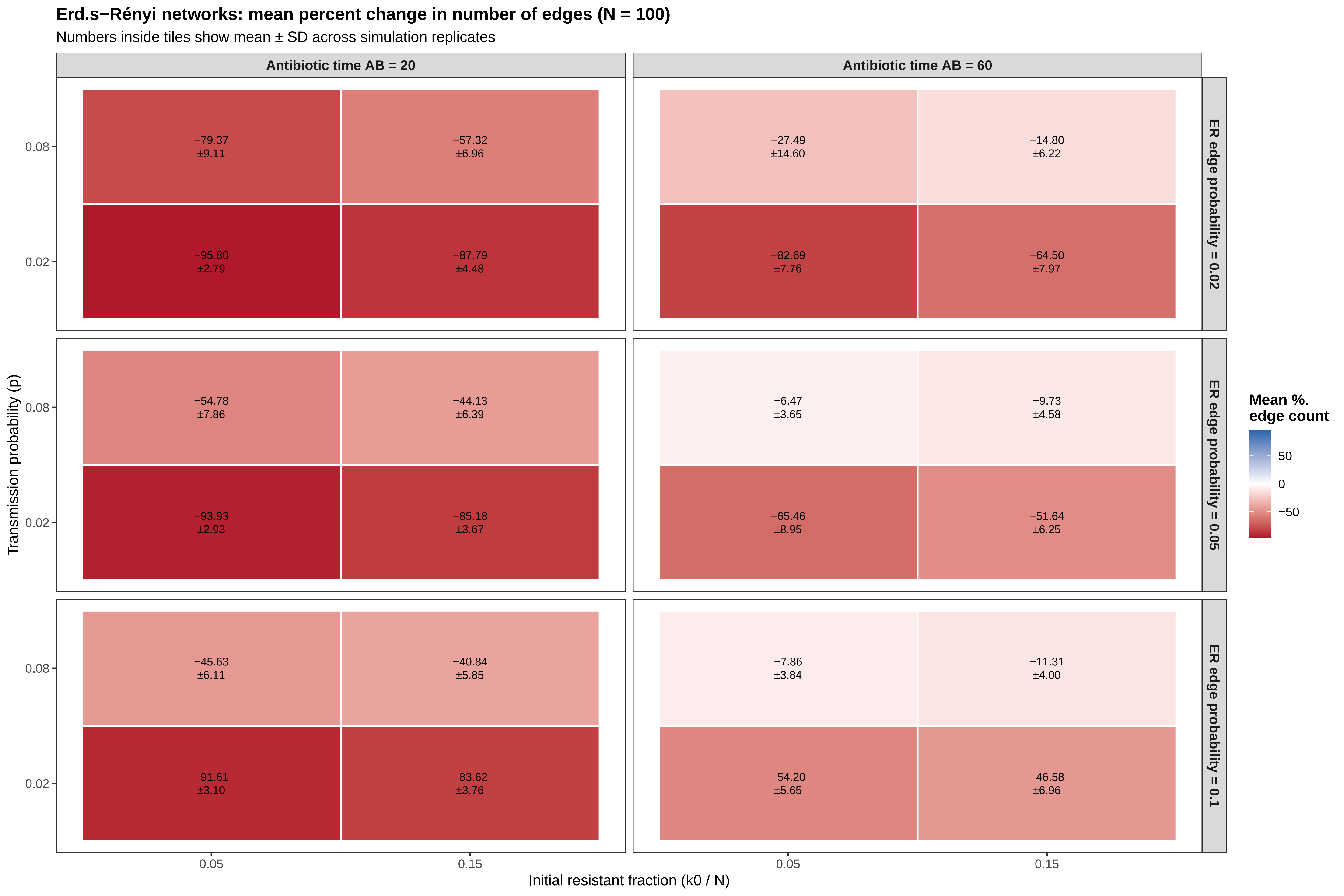

Erd.s−Rényi networks: mean percent change in number of edges (N = 100)
Numbers inside tiles show mean ± SD across simulation replicates
Antibiotic time AB = 20
Antibiotic time AB = 60
ER edge probability = 0.02
ER edge probability = 0.05
ER edge probability = 0.1
Transmission probability (p)
Initial resistant fraction (k0 / N)
0.08
0.02
0.05
0.15
−79.37 ±9.11
−57.32 ±6.96
−95.80 ±2.79
−87.79 ±4.48
−27.49 ±14.60
−14.80 ±6.22
−82.69 ±7.76
−64.50 ±7.97
−54.78 ±7.86
−44.13 ±6.39
−93.93 ±2.93
−85.18 ±3.67
−6.47 ±3.65
−9.73 ±4.58
−65.46 ±8.95
−51.64 ±6.25
−45.63 ±6.11
−40.84 ±5.85
−91.61 ±3.10
−83.62 ±3.76
−7.86 ±3.84
−11.31 ±4.00
−54.20 ±5.65
−46.58 ±6.96
Mean %. edge count
50
0
−50
